# Supplementary figures and images for: Revisiting the Injury Mechanism of Goat Sperm Caused by the Cryopreservation Process from a Perspective of Sperm Metabolite Profiles
Source: Int J Mol Sci. 2024 Aug 22;25(16):9112. doi: 10.3390/ijms25169112 (PMC11354876; doi:10.3390/ijms25169112)

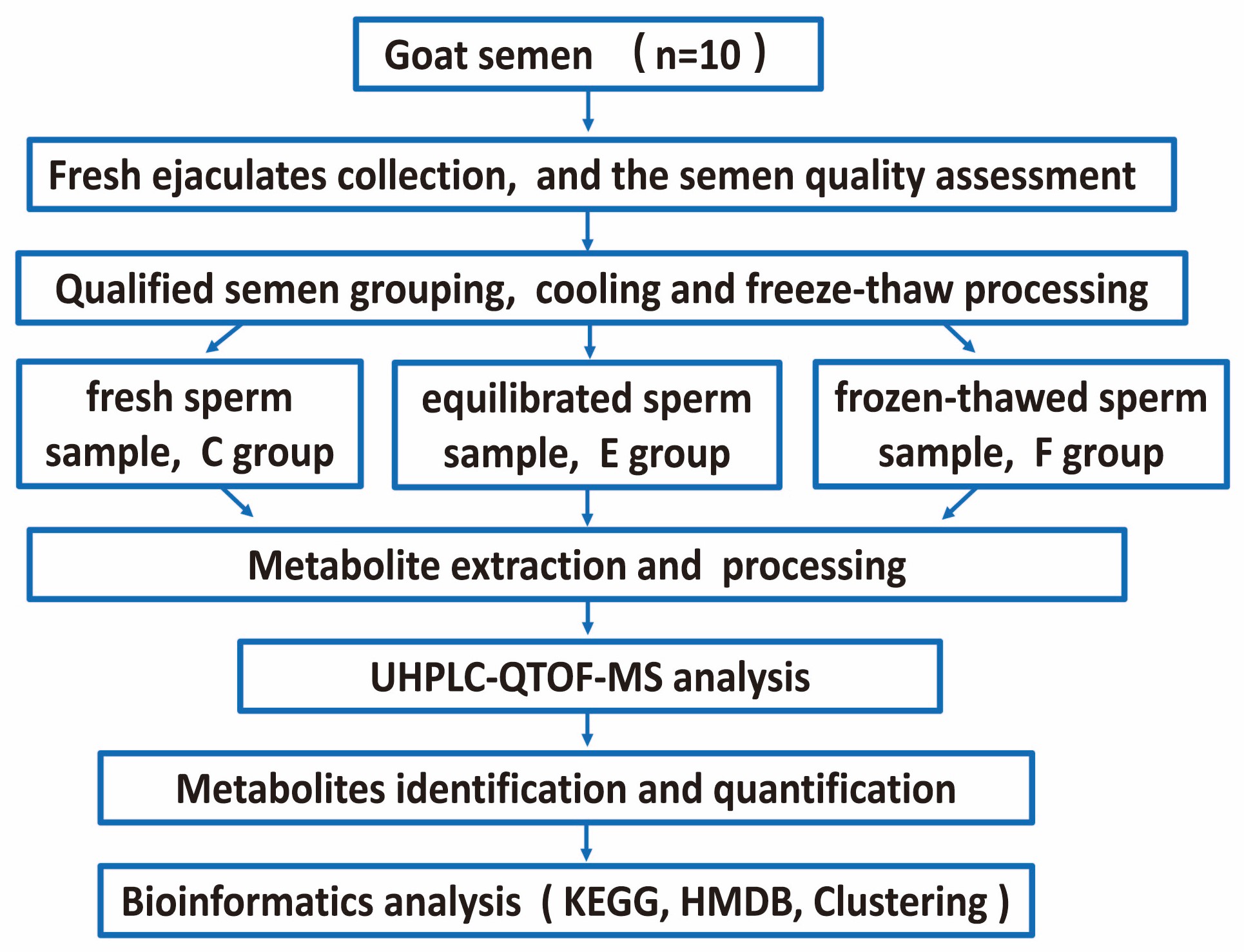

Supplement: Supplementary file 1 [file ijms-25-09112-s001.zip › Figure S1.Experimental design flow chart of this research.jpg]
